# The psychosocial impact of male infertility on men undergoing ICSI treatment: a qualitative study

**DOI:** 10.1186/s12978-024-01749-6

**Published:** 2024-02-19

**Authors:** Carmen E. J. de Vries, Esther M. Veerman-Verweij, Agnes van den Hoogen, Janneke M. de Man-van Ginkel, Henriëtta D. L. Ockhuijsen

**Affiliations:** 1 Department of Reproductive Health, University Medical Center Utrecht, 100 Heidelberglaan, 3584 CX Utrecht, The Netherlands; 2 Department of Cardiology and Cardiothoracic Surgery, Erasmus University Medical Center, 40 Dr. Molenwaterplein, 3015 GD Rotterdam, The Netherlands; 3 Department of Nursing Science, University Medical Center Leiden, 2 Albinusdreef, 2333 ZA Leiden, The Netherlands

**Keywords:** Qualitative research, Male infertility, Psychosocial functioning, ICSI

## Abstract

**Background:**

Male infertility is in 20–70% of cases the cause of a couple’s infertility. Severe forms of male infertility are best treated with Intracytoplasmic Sperm Injection (ICSI). The psychosocial impact of infertility and ICSI on men is unclear because the focus is socially, clinically, and scientifically on women. However, there is evidence that it can affect the psychological well-being of men, but these studies are mainly quantitative. Qualitative research needed to explore the experiences of infertile men in-depth is limited. Therefore, the objective of this study was to clarify the psychosocial consequences of male infertility on men undergoing ICSI to understand their experiences with reproduction problems more comprehensively.

**Methods:**

In this generic qualitative study, men who were undergoing or had undergone ICSI after a male factor infertility diagnosis were included. A purposive sample with maximum variation was sought in a fertility clinic of one university medical centre in the Netherlands. Data were collected through individual face-to-face semi-structured interviews. Thematic analysis was used to identify themes from the data.

**Results:**

Nineteen Dutch men were interviewed. The mean duration of the interviews was 90 min. *An everyday contributing backpack* was identified as the main theme, as men indicated that they always carried the psychosocial consequences of infertility and ICSI with them. *Different world perspective, Turbulence of emotions, Changing relation,* and *Selective sharing* were the psychosocial consequences that men were most affected by*.* Moreover, men indicated that they were *Searching for contribution* during ICSI because the focus was entirely on the woman.

**Conclusion:**

Men with male infertility experience psychosocial problems due to infertility and ICSI treatment. Healthcare professionals need to recognize the impact of infertility on men and create room for a role for them during ICSI.

## Plain Language Summary

Infertility, defined as not being able to get pregnant after at least 1 year of unprotected sex, is often caused by a male problem. ICSI (Intracytoplasmic Sperm Injection) is the suggested treatment for couples dealing with male infertility. During this treatment, in the laboratory, a single sperm is injected into an egg of the woman. This procedure can create an embryo that can be transferred into the woman’s uterus. Women, unlike men, have to undergo many medical examinations and treatments during ICSI which can cause changes in their mental well-being. How and whether men’s psychological and social well-being can be affected by infertility and ICSI is unclear. Therefore, by interviewing 19 Dutch men dealing with male infertility who were undergoing or had undergone ICSI, an attempt was made to better understand reproductive problems from their perspective. The interviews were all face-to-face and had a mean duration of 90 min. We found that men experienced various psychosocial consequences from infertility and ICSI: they started looking at the world differently, their relationship with their partner changed, emotions alternated, information sharing became selective and they were searching for contribution. The main theme: *An everyday contributing backpack* was identified, as men indicated that they always carried these psychosocial consequences with them in their daily lives. From this qualitative research, it has become clear that men dealing with male infertility experience psychosocial problems due to infertility and ICSI treatment. Healthcare professionals need to recognize the impact of infertility on men and create room for them.

## Introduction

Infertility affects a significant proportion of couples and is an increasing worldwide reproduction problem [1, 2]. Infertility is defined as the inability of a sexually active, noncontraceptive couple to conceive after 1 year [3]. Worldwide, approximately 15% of couples are dealing with infertility [4]. Male infertility is the cause in 20–70% of these cases [5]. Male infertility is usually the result of deficiencies in the semen concentration, morphology, or motility [6, 7]. These deficiencies can occur due to various reasons, from genetic mutations, lifestyle factors to medical diseases [8]. Although the cause of male infertility cannot always be treated, development of assisted reproductive technology has made offspring possible for these men [9, 10]. While previously only insemination with donor sperm and In Vitro Fertilization existed [11], since 1992 Intracytoplasmic Sperm Injection (ICSI) is the suggested treatment for severe forms of male infertility [12–15]. In this treatment, in the laboratory, a single sperm is injected into a mature oocyte [13].

It is important to acknowledge that infertility concerns both man and woman in a couple [16]. However, there is a social belief that infertility is a feminine issue [17], as a result of which women are conceptualized as the centre of infertility [16]. Men, by contrast, are seen as fertile appropriate to their masculinity, and are often stigmatized [18, 19]. This asymmetry can also be seen in the treatment of (male) infertility [20]. Women have to undergo most of the medical investigations and are the focus of healthcare professionals [20]. During ICSI, women have to inject hormones and undergo oocyte retrieval, which can involve heavy physical strain [21, 22]. An important role of men is submitting a semen sample [23]. Occasionally, when sperm cells are missing from the semen, men also have to undergo invasive procedures such as Testicular Sperm Extraction (TESE) [23]. Although men are the main cause of infertility in a large number of cases [24], research in the field of infertility is also primarily focused on women [16, 25].

While infertility and discomfortable infertility treatments such as ICSI can lead to depression [26], isolation [26, 27], and hopelessness [26, 28] in women, evidence on the psychosocial impact of infertility on men remains limited and inconclusive. Studies conducted in the 1990s showed that men are psychologically less affected by infertility than are women [29, 30], while more recent studies showed that the impact is nearly balanced, suggesting that infertility also affects men’s psychological well-being [31–33]. Specifically, quantitative studies have shown that men with a male factor infertility diagnosis reacted more emotionally to infertility than men from couples with another cause of infertility [34, 35]. For example, a cross-sectional study showed that infertile men experience a poor quality of life [36]. Another cohort study [37] and cross-sectional study [38] have found an association between male factor infertility and poorer sexual performance in men.

Studies with an in-depth exploration of men’s psychosocial experiences regarding infertility are underrepresented [39, 40]. One qualitative study on male infertility in men who were already fathers described feelings of grief and guilt because the men realized they were the cause of the couple’s infertility [41]. Other qualitative studies, which included men with a male factor infertility diagnosis as well as men with other causes of infertility, described infertility as a life crisis [42], with a major impact on gender identify [43, 44] and social relationships [42]. Qualitative studies on men’s experiences during infertility treatments described feelings of inferiority and exclusion [39, 45]. However, these studies did not specifically focus on a combination of male factor infertility and ICSI, while it is expected that the experiences of these men may be different as they are the cause of a problem for which their partner is undergoing treatment.

Although there is a growing body of literature on male infertility, this has primarily focused on quantitative questions [34–38]. Qualitative research needed to explore the psychosocial impact of infertility on men in-depth is scarce or focused on broad populations. Still, it is one of the top 10 prioritized research questions, according to a recent systematic review [46]. Therefore, this study aims to clarify the psychosocial consequences of male infertility on men undergoing ICSI. The findings may give a more comprehensive understanding of their experiences with reproduction problems and help improve infertility care.

## Methods

### Design

A generic qualitative study was performed to be able to explore the emic perspective of men undergoing ICSI [47–49]. A generic approach is not guided by an established set of philosophic assumptions and was appropriate for the aim of this study [50–52]. Reporting of the study was based on the consolidated criteria for reporting qualitative research (COREQ) [53].

### Participants

The target population consisted of men who were undergoing or had undergone ICSI due to a male factor infertility diagnosis. Men were considered eligible when they were able to speak and understand the Dutch language, and did undergo ICSI after 2017. A long period for recruitment was used, from 2017 until 2022, because previous studies have shown that it is difficult to recruit men with fertility problems.

Congruent with qualitative sampling methods a purposive sample with maximum variation in ethnicity, number of infertility treatments, and number of biological children was sought. With purposeful sampling, information-rich cases were deliberately selected to ensure greater understanding of the subject under study [54]. Maximum variation allowed considering the phenomenon from different angles [52]. The above-mentioned factors for maximum variation were chosen because these can influence infertility experiences [55–57]. In view of a heterogeneous sample, a sample should consist of 14–20 participants [51].

### Procedures

An independent data manager identified and selected eligible men from the electronic medical records of a fertility department of a Dutch university medical centre using a query based on factors for maximum variation. The principal investigator (HO) sent a short recruitment note by post to all selected men, asking whether she could contact them about participating in the study. Interested men could give permission by sending an email or with a reply envelope. Reminders were sent once after 6 weeks to men who had not responded. HO called the men who gave permission and provided verbal information. Next, the executive researcher (CV) sent written information about the study and called back the men to confirm their participation and schedule an interview. Signed informed consent was obtained prior to the interview.

### Data collection

Socio-demographic characteristics were collected from the electronic medical records and through the interviews. These characteristics included: age, educational level, job, ethnicity, number of infertility treatments, number of biological children, and duration of relationship.

Given the sensitive nature of the subject, men’s experiences were explored through individual face-to-face, semi-structured interviews [47]. All interviews were conducted by the first author (CV), who is a female nurse and nurse scientist not working in the fertility department. To strengthen interviewing techniques [47, 58], she first conducted two pilot interviews and evaluated these with the principal investigator (HO) [58]. Data from the pilot interviews were included in the analyses because, according to the principal investigator, sufficient depth was achieved.

An interview guide based on existing literature [39, 42–44] and expertise was used to shape the interviews. The interview guide was adapted on the basis of the pilot interviews and findings that emerged during data collection and analysis [47, 52]. The main topics covered in all interviews were: diagnosis, treatment, psychological, social, and coping (Table 1). Open-ended questions, prompts and probes were used to elicit more detailed information from participants [47]. A summary of the main topics discussed was given during the interviews as a form of member validation [58]. After the interviews, methodological and observational memos were made. Methodological memos helped to reflect on the role as researcher [47]. Observational memos were used to specify the context [47].

**Table 1:** Overview of the Interview guide

Main topic/subtopic	English question
Introduction	How are you doing?
Diagnosis	How did you experience the moment you heard that you have fertility problems?
Treatment	How did you experience the treatments you received for the fertility problems? At what point did you start thinking about treatment options? What treatments did you undergo? How did you experience the care during the treatments?
Psychologically	What was your state of mind when you were told that you have fertility problems? And how was this during the treatment?
Emotions	What emotions played a role during the diagnosis/treatment?
Behaviour	How did you behave during this period? Has it changed?
Identity	How did the diagnosis affect you as a person?
Life purposes	Did it make you look differently at things in the world? If so, can you give an example?
Social	How did you experience contact with others during the period when you were told that you had fertility problems? And how was it during the treatment?
Work	How was work during this period?
Social activities	How did you deal with social activities during this period?
Relations	Do you talk to others about the fertility problems? Why or why not? How do they react? How do you feel when people ask about your situation?
(Sexual) relation with partner	How did you experience the relationship with your partner during this period? When/how did you support each other? What were difficult moments? How was your intimate/sex life during this period?
Society/stigmatization	How do you think society views male fertility problems? How do you experience that?
Marginalization	How do you feel in relation to men of your age who do not have fertility problems?
Coping	How did you deal with the situation? What helped you? What did not? How do you get support or strength?
Concluding remarks	Are there other things that have not been covered that you would like to share? I would like to thank you for your participation

Depending on the men’s preference, interviews took place at their homes or the university medical centre. All interviews were audio-recorded. The aim was to conduct interviews until data saturation was reached [47]. Given the heterogeneity of the sample, saturation was expected to occur after 12–15 interviews [52, 59, 60].

### Data analysis

Socio-demographic characteristics were processed using SPSS Statistics for MacBook, version 27 (IBM Corp., Armonk, N.Y., USA). Categorical data are summarized using absolute and relative frequencies. Continuous data are presented as mean with standard deviation (SD) or median with interquartile range (IQR). Software program MAXQDA 12 Standard supported the analysis of the interviews [52, 58].

Interviews were analysed using the inductive thematic analysis of Braun and Clarke because it is a flexible, widely applicable method [61]. It consists of six phases [61]. First, CV transcribed the interviews verbatim and anonymised them. When transcription was complete, the audio recordings were deleted from the recording equipment. The transcripts were read and reread by CV, HO, and EV to familiarise themselves with the data [61]. During reading and re-reading, thoughts were noted, and important fragments were marked. Important fragments were those that could be used to answer the research question. In a second phase, initial codes were assigned to important fragments [61]. The meaning of the fragments was determined using observational memos. Once initial codes were generated, they were sorted into more abstract themes in a third phase [61]. In a fourth phase, themes were refined by comparing them with the coded extracts and the entire data set [61]. In the fifth phase, the essence of each theme was determined, and themes were named and defined [61]. Examples and excerpts were sought to clarify the themes in phase six [61]. The thematic analysis was an iterative process, in which the researchers constantly moved back and forth between the phases [61]. To guarantee constant comparison, researchers also moved back and forth between data collection and analysis [47, 52]. All six phases of thematic analysis were completed for each interview until data saturation was achieved. From then on, manual transcription was replaced by listening to the audio-recording by two researchers (CV and HO) independently. All subsequent phases remained the same.

During the analysis, research triangulation was applied, which in this study meant that three researchers were involved and made decisions on developing codes [47, 52, 58]. Research triangulation ensured a coding process that was better protected against interpretation bias [47, 52]. For all interviews, HO went through steps one to six of the analysis independently of CV. The second author (EV) did this for the first six interviews. Except for transcribing and anonymizing, HO and EV completed all steps. CV, HO, and EV met after every four interviews to agree on upcoming themes. When no consensus was reached, an independent researcher (AH) was involved.

An audit trail, a detailed logbook of all interpretations and choices made, was kept during the coding process [58]. AH was asked to critically review the audit trail and assess whether the interpretations and findings were supported by the data.

### Trustworthiness

To guarantee the trustworthiness of research conclusions principles of Lincoln and Guba were applied [62]. These principles were aimed at increasing the credibility, transferability, dependability, and confirmability of the study [62].

Credibility refers to the degree of correspondence between a participant’s social reality and the representation by the researcher [62]. The credibility was ensured by taking enough time to build up rapport and create a non-judgemental atmosphere during the interviews [62]. Moreover, a form of member validation was applied to check whether the information obtained from participants was correct [62]. Research triangulation and peer debriefing enhanced both credibility and conformability [62].

Transferability refers to the possibility of applying findings in other contexts [62]. By describing the participants and research process in detail, an attempt was made to accommodate the transferability [62].

Dependability and confirmability refer to whether the findings are consistent and repeatable [62]. Dependability and confirmability were guaranteed through an audit trail, triangulation of researchers, and reflexivity using memos [62].

## Results

Between January 2022 and January 2023, 60 men from the query were selected and invited to participate. Of these, 39 did not respond, one withdrew after calling for no reason and one was too ill to participate. In total, nineteen men participated. After fifteen interviews, data saturation was reached. The last four interviews confirmed data saturation.

Fifteen men were interviewed at home and four at the university medical centre. The interviews had a mean duration of 90 min (range, 58–163 min).

All men had a Western ethnicity and were born in the Netherlands. The age ranged from 30 to 45 years with a mean of 36 (SD 3.76). Six men had no children (31.6%), and the partner of three men was pregnant at the time of the interview (15.8%). All men had only undergone ICSI as infertility treatment. The number of ICSI treatments varied from 1 to 5 with a median of 2 (IQR 2) (Table 2).

**Table 2:** Socio-demographic characteristics of participants (n = 19)

Participant	Age (years)	Educational level^A^	Job^B^	Duration of relationship (years)	Children (n)	Pregnancy^C^	Infertility treatments (n)^D^	Year of last treatment
1	37	High	White	15	0	Yes	2	2021
2	38	High	Grey	9	2	No	2	2020
3	33	High	Grey	15	0	Yes	1	2022
4	41	High	White	19	1	No	3	2018
5	33	High	White	16	0	No	3	2022
6	41	High	White	9	1	No	3	2022
7	37	Middle	Blue	21	0	No	1	2022
8	31	High	Grey	9	1	No	5	2020
9	34	Middle	Blue	12	1	No	3	2019
10	36	High	Grey	8	1	No	4	2022
11	38	High	White	20	3	No	4	2019
12	45	High	White	10	0	Yes	2	2021
13	35	High	Grey	4.5	0	No	2	2022
14	37	High	White	16	2	No	4	2020
15	32	High	White	7	1	No	1	2021
16	35	High	White	6.5	1	No	2	2020
17	31	High	White	6	1	No	1	2021
18	30	High	White	8.5	1	No	1	2020
19	37	High	White	5	1	No	2	2020

^A^ Low refers to elementary education, middle refers to high school or middle-level applied education, and high refers to higher professional or academic education; ^B^white-collar jobs refer to administrative and management positions, blue-collar jobs refer to manual labour, and grey-collar jobs are a combination of white and blue-collar jobs; ^C^yes if partner was pregnant at the time of the interview; ^D^ICSI treatments

**Fig. 1:**
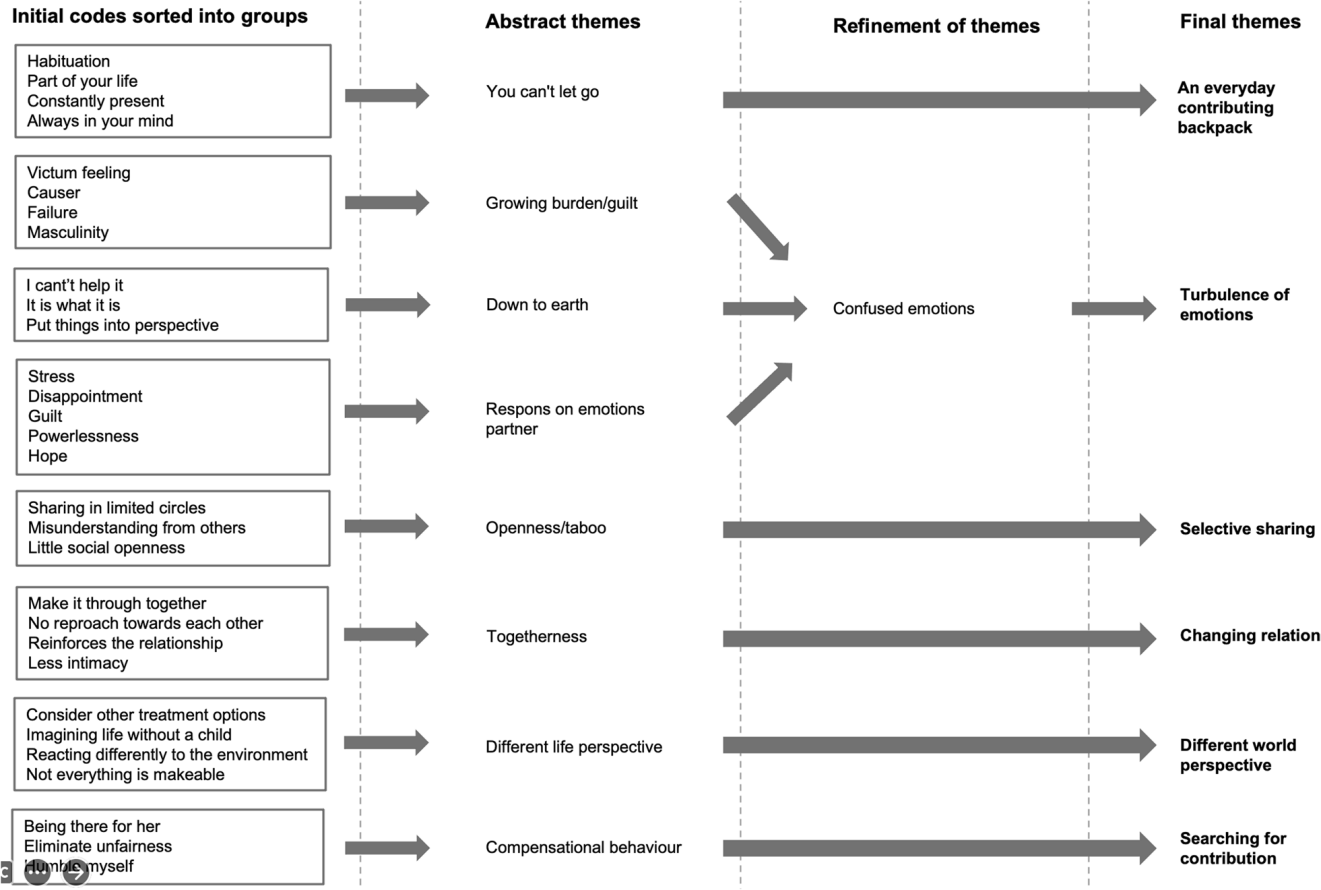
The development from codes to themes

From the analysis, 50 initial codes emerged, which were reduced to 30 after several rounds of consultation. The 30 initial codes could be divided into eight themes by grouping them. Refinement of the eight themes resulted in six final themes which were thoroughly named and defined (see Fig. 1 for the development from codes to themes). One of the six themes is an overarching theme: *An everyday contributing backpack*. The other five themes identified are related to the overarching theme: *Different world perspective, Turbulence of emotions, Searching for contribution*, *Changing relation,* and *Selective sharing*. The overarching theme reflects that the men always carried the psychosocial consequences of the diagnosis and treatment with them in their daily lives:

*“It did have an impact, yes. At times I was able to let it go, but it was a central part of my life. It was, though, something we both talked about a lot and yes, you are confronted with it every day. It is quite a big part of your life.” (Participant 17)*

Infertility and ICSI were always in the back of their minds, but still, they were able to live on. The five related themes reflect the psychosocial consequences the men were most affected by in their lives.

### Theme 1: different world perspective

Most men reported changes in their world perspective after being diagnosed with infertility. They realized that the world was not ‘makeable’. Men never had considered that having a child would be difficult because for many of them everything in life up to that point seemed obvious:

*“You always thought at some point to have children, but yes that’s not true. [Laughs]. That’s a little more complex. Then you find out: oh well it can also be like this. You don’t just go to bed with each other a few times and you try it for a few months and then you’re pregnant.” (Participant 2)*

Some men were more grateful for things they did have, such as family or friends. Other men had become more cautious toward others, for example in making comments about having children:

*“You are going to approach people very differently. You are more careful what you, what you say, yes you do that, yes you do. You become, maybe you become, yes a little more respectful towards people maybe.” (Participant 3)*

The way men viewed pregnant women or people with children often changed as well. Some men experienced jealousy, unfairness, and annoyance. These men often avoided confronting pregnant women and children because it was too painful:

*“If we had a day out where the kids came along, for example, then, then I would pass. Because then I had, I had at that moment, I really did not feel like it. Because that was just yes quite confronting actually.” (Participant 3)*

Other men were not bothered by this and sometimes gravitated more toward pregnant women and children. One of them called it ‘wallow’, to be able to experience it too.

Many men indicated that the length and progression of the ICSI process changed their perspective toward infertility treatments. While infertility treatments had previously seemed far away and unknown, many men started to consider treatment options such as adoption after the diagnosis. Men who had just started ICSI or had already had a successful ICSI indicated that they only discussed treatment options with their partners. Other men, for whom the ICSI process was arduous or unsuccessful had often imagined or even accepted a life without children:

*"And the funny thing is also, what I said: if I would have said two years ago that we would talk about donation material at all, I was really like: why on earth? But now we've moved on and noticed that it's not so obvious. Suddenly it's open for discussion and it's becoming a real possibility. So that also makes a difference where you stand in this whole course and how difficult or easy, what setbacks you've already had." (Participant 5)*

### Theme 2: turbulence of emotions

Men experienced varying emotions from the time they were diagnosed with infertility. Many men felt sad because they thought their future was going to fall apart. Some men reported that the diagnosis affected their self-esteem. They said it felt like a personal failure or a violation of their masculinity:

*“Yeah that's, yeah just kind of painful. You always think that your status or that your masculinity depends on your reproductive ability. That's your primitive brain that thinks that. It feels like a kind of failure but actually it's something you can't do anything about.” (Participant 8)*

In contrast, other men were level-headed and tried to put the situation into perspective:

*“… I see people around me who are ill or whatever and then I think: yes that is much worse in my eyes... And that's not to say that it's much easier but yes that's how I kind a look at it.” (Participant 9)*

The moment ICSI was offered as a treatment option, men experienced mostly positive emotions such as happiness, relief, and hope. One man said that it felt like winning a race. For many men, the positive emotions quickly turned into feelings of guilt and powerlessness when they realized that it was the partner who had to undergo the medical procedures during ICSI. Men spoke of ‘being the cause’, ‘being worthless’, and ‘not being able to fulfill their part’.

Some men noticed that their feelings of guilt increased if the ICSI process took longer:

*“There is, after all, a kind of guilt feeling… When you see all the needles, injections, hormone treatments and other heavy shit… During the course, I started feeling more and more guilty about it. I thought yes, shit, you know, I have a problem and you have to endure it all. Because at the end of the day, it's pretty intense, you know, just putting injections in your stomach for weeks..., the growth of eggs on the ovaries, it's just all extremely intense.” (Participant 4)*

The guilt and powerlessness put pressure on the men when they had to submit semen, because in their eyes that was the only thing they had to do:

*“That was my only concern, that you provide enough material so that they can do an ICSI. Because let’s be honest, my wife had to inject all those hormones…, so I have to do something in return. That was actually the only pressure I felt. I found that a bit exciting: will it work on that day?” (Participant 2)*

Men indicated that they tried to keep hope and remain positive. However, this was not easy; some suffered from feelings of insecurity, disappointment, and frustration. Men said that these feelings intensified as the ICSI process was more difficult or took longer:

*“…With the second ICSI it didn’t work out and we went round after round after round of trying, disappointment, trying, disappointment and yes if you just have to inject hormone extremes in your body, that became both physically and mentally harder and harder. That burden became greater and greater… that guilt, on my side, became greater and greater. Like yes: when does this stop? [Short silence]. So, that agony got bigger and bigger, still because the problem was with me.” (Participant 4)*

### Theme 3: searching for contribution

Most men wanted to eliminate their feelings of guilt, powerlessness, and unfairness that arose from the realization that their partner had to undergo all medical treatments. Therefore, men began to search for their own contributions. For instance, some men were modifying their lifestyles to produce better semen. Other men began to exhibit compensatory behavior to relieve their partner, such as paying more attention to the partner, taking over tasks from the partner, but also humbling themselves:

*“…I was exhibiting some compensatory behavior anyway… Okay, then I need to be there more in other areas. I need to be kinder, more attentive, and supportive in other in areas… I was going to give up on more things myself… So I started to put myself more in the background.” (Participant 4)*

Many men reported that they could do no more than ‘being there’ for the partner:

*“I just have to accept it because that’s just how it goes, that’s the situation, that’s how the process goes or how the treatment goes, there is not much you can do. [Short silence]. Then you can’t do more than be there for your wife, that is the only thing you can do.” (Participant 2)*

However, some men indicated that their search for recognition and contribution was hampered by the central focus on the woman in the hospital. These men said that they were literally sidelined and could not feel part of the process. For some of them, this caused frustration:

*“There is no concern for the man, absolutely not. It's just about the woman. ... That was literally said by the caregiver.’’ (Participant 14)*

*“The woman is addressed; the man sits next to her. If something is said, the woman is looked at directly... The man is involved very little. I've noticed that more and more… That the man has only one task at that moment, he goes into a room, he does his thing and that's it. Just ask: “How do you experience it? Do you have any questions? That was not even done.” (Participant 10)*

Others thought that it was more than logical:

*“Everything in the hospital made me realize that it’s not about the man, it’s about the woman. Yes of course because that is where the eggs are generated, that is where they are retrieved, she goes through all the treatments and the hormones, so I have nothing to do with that.” (Participant 12)*

### Theme 4: changing relation

The interviews revealed that men experienced positive and negative changes in their relationships with their partners. In terms of positive experiences, men said that the relationship became stronger and more profound because they saw the diagnosis as a joint problem that they had to solve together:

*“You have a common enemy and you’re attacking it together, well that is, that is, that’s what makes every relation better.” (Participant 1)*

Although the relationship became stronger, some men mentioned that their partners wanted to discuss the possibility of ending the relationship. These men understood that having a child with another man would be easier, but none of them were afraid that their partner would really leave:

*"Yes, it is confronting that I am infertile… Yeah and then you just feel very powerless. I can't change it so if she really, really wants a child and it has to go that way, yes, then this is what you have to do. But on the other hand, I have trust in our relationship and in her, she's not going to leave me for this I know that, that is the funny thing, strangely enough. I don't see that happening [Laughs]. So, I wasn't worried about her really considering it…" (Participant 5)*

In contrast to the positive strengthening of the relationship, men experienced deterioration in terms of intimacy. Almost all men reported that sex was a purposeful act around the time of the diagnosis. Men described it as: ‘medical act’, ‘planned’ or ‘a must’. They had sex less frequently and experienced it as less romantic. Men planned sex because they were hopeful that it would still work out naturally:

*“Yes, just a lot more regular, well just try to time it. You just know your cycle: now we are after ovulation, so it makes no sense at all anymore. Yes, yes hope at the beginning of a cycle, it became more regular. I just feel pressure like: yes, I just really have to do something now, you do it purely because you think you can do something.” (Participant 8)*

### Theme 5: selective sharing

All men consciously chose to whom they would share information and what information they shared about the diagnosis and ICSI. Few men chose to isolate themselves and discuss the situation only with their partner. Other men chose to share it with all their family and friends.

However, most men shared information with a limited circle of family and friends they trusted. There was also variation in what information was shared. Some men shared almost everything, others kept it superficial and, for example, did not tell that they were the cause. Reasons for openly sharing included: ‘relief’, ‘recognition’, and ‘support’. Reasons for not sharing information were: the topic was private and intimate, feeling anxious to share disappointment, and being ashamed of the diagnosis. Men noted that they talked more openly about the situation if the ICSI process took longer:

*“Well, in the beginning, I was more closed-minded about it, so I just didn't want anyone to know about it and so on, and then you become isolated. But actually, it is quite nice to talk about it with people, because then you can get it off your chest, so it is only nicer for yourself. But in general, I am more of keeping it to myself and I will solve it. I've really learned that that's not useful at all. So yes in the beginning isolation, yes it is a kind of secret that you carry with you.” (Participant 8)*

When men shared information about their infertility, they discovered that many more men suffered from the same problem. All men reported that it was pleasant to realize that they were not the only ones. They liked being able to connect with peers in this way:

*“…Where you normally wouldn't know or, or don't hear so much about it, then suddenly when you then tell it yourself, then it almost seems like the whole world around you has almost, also been in such a situation.’’ (Participant 6)*

## Discussion

In this study, the aim was to explore the psychosocial consequences of male infertility on men undergoing ICSI. One overarching theme was identified: *An everyday contributing backpack*, with five related themes: *Different world perspective, Turbulence of emotions, Searching for contribution, Changing relation*, and *Selective sharing*.

Since the literature on male infertility is limited, it is difficult to compare our findings. However, some of our themes were found in the general literature on infertility. For example, men in our study indicated that the diagnosis of infertility made them realize that the world is not makeable, as part of the theme *Different world perspective*. This seems to be related to loss of control, a familiar theme in studies of women’s experiences with infertility [63, 64]. This consistency reinforces our results and may indicate that our themes are relevant in the current field. However, we will emphasize themes not found in the qualitative literature on infertility or that were more prominent in our study.

First, themes that were similar to our overarching theme were not found. This can be explained by the fact that previous research almost never included infertile men, who are precisely those who suffer from a backpack of psychosocial consequences since they are the cause of the problem for which the partner must undergo treatment.

Second, we also found no literature underpinning our theme *Turbulence of emotion*s. Previous qualitative studies mention aspects such as loss of masculinity and disappointment [43, 65, 66], but do not mention the alternation between sadness, happiness, and guilt. These alternating moods we found could be explained by the theory of stress and coping of Lazarus et al. which assumes that every person makes individual and continuous cognitive efforts to encounter events that are appraised as exceeding their resources [67, 68].

Third, men in our study were *Searching for contribution* during ICSI because they felt guilty. Seeking contribution and unburdening the woman are strategies reported in previous qualitative studies involving men [69–72]. These studies, however, did not all focus on men with a male factor infertility diagnosis. This can explain why this theme was more prominent in our study. It reinforces our suspicion that men who are the cause of infertility suffer more from guilt and compensational behavior. A recent systematic review found evidence that men who are the cause of infertility indeed have more intense emotions than men from couples with other causes of infertility [73]. Many men in our study found themselves side-lined and unable to contribute to ICSI. Findings from previous research and our findings highlight the importance of involving men during treatment [74–77].

Fourth, we found that the sexual relationship between man and woman worsened and that some couples considered ending the relationship*.* It is notable that qualitative studies on infertility hardly addressed these topics, whereas both have been addressed in quantitative studies [78–81]. Maybe men find it easier to mention intimacy issues in quantitative studies where they are more anonymous. The quantitative studies lack, however, detail as obtained in our study.

Fifth, *Selective Sharing* was reported in one large qualitative study. This study was conducted among infertile men in India undergoing various infertility treatments (including ICSI) [82]. These men said that they shared information only with their partners, indicating that there may be ethnic and cultural differences regarding the experiences of infertile men. The study by Schick et al. described selective sharing as a small component of their study [69]. Presumably, it was a larger component in our study because the participants themselves were the cause of the problem and therefore wished to have more control over sharing of information.

A strength of this study is that interpretation bias was reduced because three researchers independently performed the coding process. In addition, the preconceptions of the first author were limited because she works in a different field. Moreover, by applying the principles of Licoln and Guba the trustworthiness of research conclusions was enhanced. However, some limitations need to be discussed. Due to the non-coercive nature of the recruiting method, it was not possible to achieve maximum variation in ethnicity. Therefore, the findings are only transferable to men with a Western ethnicity and born in the Netherlands. Another weakness was the time between the last treatment and the interview. Some men could not specifically remember their experiences, thoughts, and emotions, which might have resulted in recall bias. Men who were less able to remember their emotions might have described the trajectory more level-headed and positively than those who could remember everything in detail. Therefore, a recommendation for future qualitative research would be to only include men undergoing ICSI at the time of the study. However, further research can focus on various facets of male infertility, as there are many gaps in the literature. Qualitative research could focus on participants of different demographic profiles, for instance, men of non-Western ethnicities. More quantitative research is needed to confirm a relationship between the duration of ICSI and psychological outcomes, as our study shows some evidence that emotions can be more intense when the ICSI process is longer.

## Conclusion

Since our results show that men with male infertility experience psychosocial problems and feel sidelined during ICSI, it is important to use these experiences to optimize infertility care. Healthcare professionals need to recognize the impact of infertility on men and create room for a role for them during ICSI. Policymakers and researchers could help to design interventions that enhance the partnership of infertile men and fulfill their contribution.

## Data Availability

The datasets used and/or analyzed during the current study are available from the corresponding author on reasonable request.

## Abbreviations

ICSI: IntraCytoplasmic Sperm Injection
TESE: TEsticular Sperm Extraction
COREQ: COnsolidated criteria for REporting Qualitative research
IBM SPSS: Statistical Package for the Social Sciences
SD: Standard deviation
IQR: InterQuartile Range
MAXQDA: MAX qualitative data analysis
